# Enzymatic Kinetic Resolution of Racemic 1-(Isopropylamine)-3-phenoxy-2-propanol: A Building Block for β-Blockers

**DOI:** 10.3390/ijms251910730

**Published:** 2024-10-05

**Authors:** Joanna Chałupka, Michał Piotr Marszałł, Adam Sikora

**Affiliations:** 1 Department of Pharmaceutical Technology, Faculty of Pharmacy, Medical Biotechnology and Laboratory Medicine, Pomeranian Medical University in Szczecin, 71-251 Szczecin, Poland; 2 Department of Medicinal Chemistry, Faculty of Pharmacy, Collegium Medicum in Bydgoszcz, Nicolaus Copernicus University in Toruń, Dr. A. Jurasza 2, 85-089 Bydgoszcz, Poland

**Keywords:** β-blockers, ionic liquid, enantioselective biotransformation, lipase, *Candida rugosa*

## Abstract

This study aimed to optimize the kinetic resolution of building blocks for the synthesis of β-blockers using *Candida rugosa* lipases, which could be potentially used to synthesize enantiomerically pure β-blockers further. Reaction mixtures were incubated in a thermostated shaker. Qualitative and quantitative analyses of the reaction mixtures were performed using chiral stationary phases and the UPLC-IT-TOF system. Of the 24 catalytic systems prepared, a system containing lipase from *Candida rugosa* MY, [EMIM][BF_4_] and toluene as a two-phase reaction medium and isopropenyl acetate as an acetylating agent was optimal. This resulted in a product with high enantiomeric purity produced via biotransformation, whose enantioselectivity was E = 67.5. Using lipases from *Candida rugosa* enables the enantioselective biotransformation of the β-blockers building block. The biocatalyst used, the reaction environment, and the acetylating agent significantly influence the efficiency of performer kinetic resolutions. The studies made it possible to select an optimum system, a prerequisite for obtaining a product of high enantiomeric purity. As a result of the performed biotransformation, the (*S*)-enantiomer of the β-blocker derivative was obtained, which can be used to further synthesize enantiomerically pure β-blockers.

## 1. Introduction

More people die each year from cardiovascular diseases than from any other cause [1]. Over three-quarters of heart disease and stroke-related deaths occur in low-income and middle-income countries. Hypertension—or elevated blood pressure—is a severe medical condition significantly increasing the risk of heart, brain, kidney, and other diseases. Hypertension can be defined using specific systolic and diastolic blood pressure levels, or the reported use of antihypertensive medications [2]. An estimated 1.4 billion people worldwide have high blood pressure, but just 14% have it under control [3]. However, cost-effective treatment options exist [4,5]. β-blockers exert their effects by binding to β-adrenergic receptors (primarily β1 and β2 receptors) on the surface of cardiac and smooth muscle cells. By competitively inhibiting the binding of catecholamines (like adrenaline) to these receptors, they prevent the activation of the G protein-coupled signaling pathway. This inhibition reduces the production of cyclic AMP (cAMP) and downstream protein kinase A (PKA) activity, leading to decreased calcium influx into cells, ultimately reducing heart rate, contractility, and overall cardiac output. Because the β-blockers in their chemical structure have an asymmetric carbon atom, their chiral center, they exist as two enantiomers, i.e., (*R*)-enantiomer and (*S*)-enantiomer. (*S*)-enantiomers of β-blockers are usually responsible for the therapeutic action, since the (*R*)-enantiomers of β-blockers have significantly lower affinity to the β-adrenergic receptors and could cause additional adverse events [6,7]. Nevertheless, β-blockers are still mainly administrated as racemates instead of pure eutomers, and thus could be responsible for unnecessary side effects [7,8]. The most common adverse effects associated with the use of β-blockers include the occurrence of fatigue, bradycardia (slowed heart rate), dizziness, cold extremities, and sleep disturbances; additionally, symptoms such as depression, impotence, and the masking of hypoglycemia symptoms in diabetic patients may also occur [9,10,11,12].

The rising demand for enantiomerically pure pharmaceuticals has driven the development of efficient, environmentally friendly synthetic methods. Kinetic resolution via enzymatic catalysis has emerged as a key strategy, with lipases gaining attention for their ability to catalyze the hydrolysis and transesterification of various substrates, producing valuable chiral intermediates. Recently, the use of ionic liquids (ILs) in enzymatic reactions has attracted significant interest. ILs are known for their non-volatility, non-flammability, and low toxicity, making them a favorable alternative to traditional solvents. They create a stable environment for enzymes, preserving the structure and activity of lipases, enhancing their efficiency, selectivity, and operational stability [13,14,15]. ILs also offer ecological benefits as “green solvents”. They reduce hazardous volatile organic compound emissions, enable safer handling, and are highly recyclable, allowing reuse in multiple reaction cycles, thus promoting sustainability in pharmaceutical synthesis [16,17,18]. Recent studies have shown that ILs positively influence the enantioselectivity of enzymatic reactions, particularly in producing chiral pharmaceutical intermediates. By improving solvation properties and dissolving both hydrophobic and hydrophilic substrates, ILs can enhance reaction rates and product yields [19,20]. This integration of enzyme catalysis with the sustainable benefits of ILs aligns with global initiatives for greener chemical processes.

**Figure 1:**
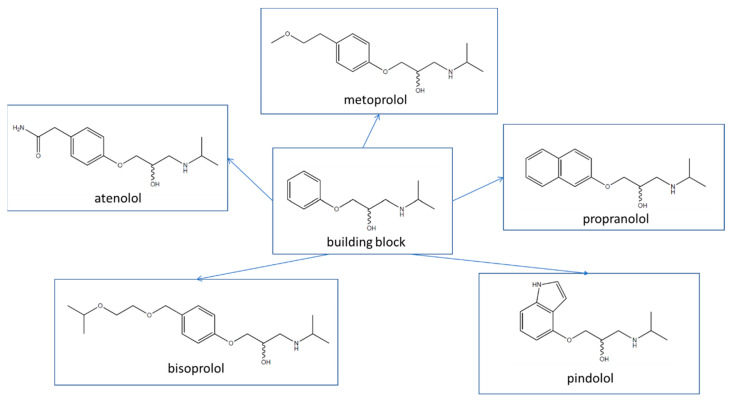
Examples of different β-blockers containing a common building block and chiral center.

In order to obtain enantiomerically pure compounds, there are various approaches. Nevertheless, the kinetic resolution of chiral compounds using lipases as enantioselective biocatalysts remains a topic of extensive research due to its significant potential for producing enantiomerically pure substances [21,22,23,24,25,26,27,28,29]. This study aimed to optimize a method for the kinetic resolution of the β-blockers building block, e.g., 1-(isopropylamine)-3-phenoxy-2-propanol, using lipases as enantioselective biocatalysts in a two-phase catalytic system consisting of toluene or dichloromethane and an ionic liquid. The enantiomerically pure 1-(isopropylamine)-3-phenoxy-2-propanol could be further utilized to synthesize various β-blockers (Figure 1). The study analyzed factors affecting the kinetic resolution, such as the type of lipase, incubation time, reaction medium, or ionic liquids used. Analyses were conducted using ultra-high-performance liquid chromatography, high-resolution ion trap mass spectrometry, and a time-of-flight analyzer (LCMS-IT-TOF). As part of the research, the influences of the factors mentioned above on the values of parameters, such as enantioselectivity, the enantiomeric purity of products and substrates, and the degree of conversion of the kinetic resolution performed, were assessed.

## 2. Results and Discussion

### 2.1. Enantioselective Biotransformation of Racemic β-Blocker Building Block

This study aimed to develop an optimal method for the kinetic resolution of the building block of the β-blocker 1-(isopropylamine)-3-phenoxy-2-propanol using lipases as enantioselective biocatalysts in a two-phase catalytic system. The kinetic resolution of the racemic mixture of the β-blocker derivative was performed by enantioselective transesterification using lipase from *Candida rugosa* occurring in two isoforms, OF and MY. Toluene or dichloromethane with or without the addition of [EMIM][BF_4_] and [EMIM][MSF_3_] were used as the reaction medium, while the acyl group donor was vinyl acetate or isopropenyl acetate. Analyses were performed using ultra-high-performance liquid chromatography coupled to high-resolution ion trap mass spectrometry, time-of-flight analyzer (LCMS-IT-TOF), and chiral stationary phases. The influences of various factors determining the efficiency and selectivity of the reaction, such as the type of lipase, the acyl group donor incubation time, the reaction medium, and the use of ionic liquids, were investigated. The study showed that the kinetic resolution under the conditions tested was reached after 24 h. Subsequent catalytic cycles resulted in substrate depletion, and the observed products did not show sufficient enantiomeric purity. Of the studied factors affecting the kinetic resolution of (*R*, *S*)-1-(isopropylamine)-3-phenoxy-2-propanol, the most favorable systems were those that contained [EMIM][BF_4_] as the ionic liquid. In these systems, it could be observed that the chromatograms obtained showed a satisfactory kinetic resolution of the racemic mixture of the β-blocker derivative into substrates and products. In addition, based on the chromatograms obtained and the peak areas of reaction systems 1–24, the values of parameters describing the enantioselectivity of the kinetic resolution taking place, such as enantiomeric excess for substrates and products, enantioselectivity (E) and conversion (C), were calculated. The compositions of tested reaction systems are shown in Table 1. The most favorable system for the kinetic resolution of racemic 1-(isopropylamine)-3-phenoxy-2-propanol was found to be a system containing isopropenyl acetate as the acylating agent, [EMIM][BF_4_] and toluene as the two-phase reaction medium, and lipase from *Candida rugosa* MY as the biocatalyst. The reactions in this configuration could be considered enantioselective, as evidenced by the high E-value of 67.45. Furthermore, this system is characterized by a high enantiomeric excess of products (ee_p_ = 96.17%), which also proves the enantiomeric purity of the obtained product.

### 2.2. Effect of Reaction Time

The kinetic resolution reaction of the racemic mixture 1-(isopropylamino)-3-phenoxy-2-propanol was carried out for 240 h. The determined reaction time of 240 h is, indeed, prolonged; however, we conducted kinetic resolution assessments at 24 h intervals throughout this period to systematically collect data on the progress of the resolution. This approach allowed us to monitor the reaction dynamics and obtain comprehensive results. The prepared samples were analyzed using ultra-high-performance liquid chromatography coupled to tandem mass spectrometry, ion trap (IT), and a time-of-flight (TOF) analyzer (LCMS-IT-TOF).

From the chromatograms obtained, it was observed that the reaction was run entirely within 24 h. During the subsequent hours of the response, substrate depletion occurred, and consequently, the observed products did not show sufficient enantiomeric purity (Table 2). The effect of loss of operational enantioselectivity after 24 h of incubation of the reaction mixture was probably due to excessive distomer biotransformation and eutomer depletion. It is therefore advisable to carry out sampling only during the first 24 h of the reaction at shorter intervals, e.g., every two hours, to accurately observe the kinetic separation reaction’s progress. Because of the above, it should be objectively stated that the reaction time is an essential factor influencing the parameters describing the enantioselectivity of the reaction and the purity of the obtained products. On the other hand, data from the literature show that each lipase has an optimal running time and specific sensitivity to activation.

### 2.3. Effect of the Biocatalysts

**Figure 2:**
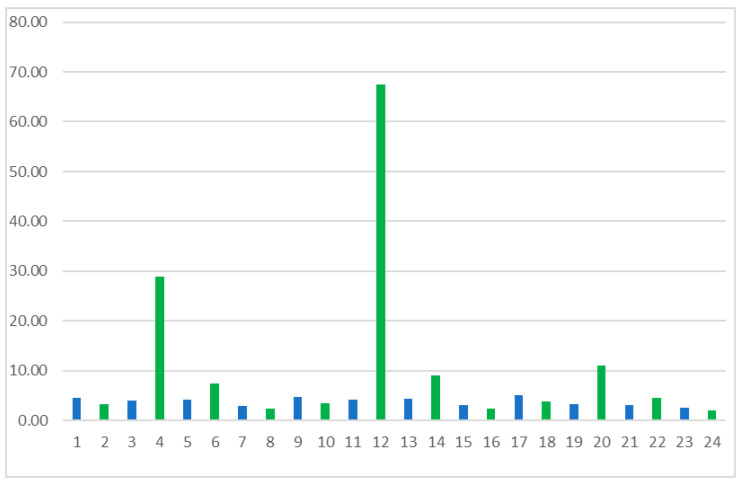
Enantioselective values for the esterification reaction of a β-blocker derivative using lipases from *Candida rugosa* OF (blue bars) and MY (green bars) depending on the reaction system’s composition.

**Figure 3:**
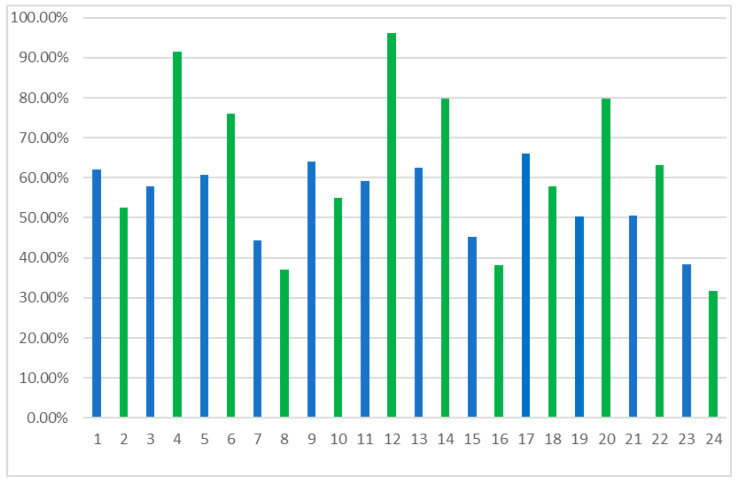
The enantiomeric excess of products for the esterification reaction of a β-blocker derivative using lipases from *Candida rugosa* OF (blue bars) and MY (green bars), depending on the reaction system’s composition.

The study compared the kinetic resolution parameters of reactions conducted using two commercially available *Candida rugosa* OF and MY lipases. In both cases, the lipases tested reacted preferentially with the (*S*)-enantiomer. While both isoforms showed wide specificity towards oils and fats, the primary difference lies in their lipase activity: *Candida rugosa* MY showed an activity of 30,000–60,000 U/g, while *Candida rugosa* OF showed a significantly higher activity of 360,000–400,000 U/g. This difference in enzymatic activity is likely related to their performances in the assay, where *Candida rugosa* OF demonstrated higher efficiency due to its greater lipolytic activity. Identical reaction conditions were used for each lipase to compare their catalytic properties, and based on the optical purities of the products obtained, defined by the enantiomeric excess of the eep products, a comparison of the activities of the two lipases was made (Figure 2 and Figure 3). Considering the results obtained, the most efficient conversion and the best enantioselectivity were achieved using MY lipase in the system with [EMIM][BF_4_], toluene, and isopropenyl acetate (No 12).

### 2.4. Effect of Reaction Medium

The effect of the reaction medium on the kinetic separation of the β-blocker derivative was the final factor tested in terms of the reaction’s efficiency. Based on preliminary studies, two organic solvents, toluene and dichloromethane, were selected for final analysis in 24 catalytic systems, which included a racemic mixture of 1-(isopropylamine)-3-phenoxy-2-propanol, lipase from *Candida rugosa* OF or MY, and vinyl acetate or isopropenyl acetate as the acetyl group donor, 16 of which additionally contained the ionic liquids [EMIM][BF_4_] and [EMIM][MSF_3_].

Based on the results, toluene was the best of the solvents tested, as a high enantiomeric excess value of the products ee_p_ = 96.2% was obtained in its presence. The choice of the appropriate solvent affects the activity of lipases, so this is a critical element in getting the most catalytically efficient system. The preliminary selection of the organic solvent as the reaction medium was made according to the literature analysis, which is fully described in the discussion section.

### 2.5. Effect of Ionic Liquids

The effect of ionic liquids on the kinetic separation of the β-blocker derivative was another factor tested to determine the reaction’s efficiency. Based on preliminary studies and literature analyses, two ionic liquids were selected for final analysis, 1-ethyl-3-methylimidazolium tetrafluoroborate [EMIM][BF_4_] and 1-ethyl-3-methylimidazolium trifluoromethanesulfonate [EMIM][MSF_3_] [30,31]. The ionic liquids were in 16 catalytic systems comprising a racemic mixture of 1-(isopropylamine)-3-phenoxy-2-propanol, lipase from *Candida rugosa* OF or MY, toluene or dichloromethane as the reaction medium and vinyl acetate or isopropenyl acetate as the acetyl group donor. Based on the results obtained, it was concluded that only the presence of [EMIM][BF_4_] had a beneficial effect on the biotransformation. In contrast, the direct addition of [EMIM][MSF_3_] to the reaction systems decreased the kinetic resolution parameters. Adding ionic liquids can significantly enhance the results of the enzymatic kinetic resolution by stabilizing enzyme structures, thereby reducing denaturation and improving enzyme durability under challenging reaction conditions. This stabilization often leads to increased enzyme activity and efficiency in the resolution process. As previously described in the literature, various ionic liquids, containing different anions and cations, can interact with the enantioselective biocatalyst through ionic and dipolar interactions, hydrogen bonding, or van der Waals forces [32]. However, it is crucial that these interactions do not adversely affect the enzyme conformation. By carefully selecting ionic liquids that provide stabilizing effects without disrupting the structural integrity of the enzyme, the overall performance and longevity of the biocatalyst in kinetic resolution can be significantly enhanced. Furthermore, ionic liquids can increase the solubility of substrates and products, facilitating better distribution and accessibility to the enzyme, enhancing reaction efficiency, and improving the enantiomers’ resolution. Additionally, it is worth noting that the created biphasic catalytic system facilitates the easy separation of products and substrates from the protein biocatalyst, providing significant advantages. This separation allows for the isolation of the individual components of the catalytic system, enabling the reuse of protein biocatalysts in other catalytic reactions. Furthermore, ionic liquids are known for not negatively affecting the operational activity of enzymes, including lipases, thus enhancing their stability and efficiency [30,33]. This characteristic makes ionic liquids an attractive option for various applications in enzymatic catalysis, ultimately contributing to more sustainable and efficient chemical processes. Additionally, ionic liquids can modify the reaction environment’s properties, such as polarity and viscosity, optimizing these conditions to influence the enzymatic reaction positively. These combined effects contribute to the more efficient and precise enzymatic kinetic resolution.

## 3. Materials and Methods

### 3.1. Chemicals

Acetonitrile, dichloromethane, methanol, isopropanol, formic acid, [EMIM][BF_4_], [EMIM][EtSO_4_], [HMIM][BF_4_], [EMIM][MSF_3_], [DMIM][MeSO_4_] and toluene were purchased from Merck (Sigma-Aldrich Co., Stainhaim, Germany).

Racemic 1-(isopropylamine)-3-phenoxy-2-propanol was purchased from Toronto Research Chemicals (Toronto, ON, Canada).

Lipases from *Candida rugosa* OF and MY were gifts from Meito Sangyo Co., Ltd. (Tachikawa, Japan).

The study obtained the water used using a Milli-Q Water Purification System (Millipore, Bedford, MA, USA).

### 3.2. Instrumentation

HPLC samples were washed using refrigerated CentriVap concentrators purchased from Labconco (Kansas City, MO, USA).

The chiral UPLC-IT-TOF system used for HPLC studies consisted of an autosampler (SIL-40AC, Shimadzu, Kyoto, Japan), two solvent feed pumps with a gradient system (LC-40AD, Shimadzu, Kyoto, Japan), a degasser (DGU-30A5, Shimadzu, Japan), a column oven (CTO-40AC, Shimadzu, Kyoto, Japan), a UV detector (SPD-M20A, Shimadzu, Kyoto, Japan) and a triple quadrupole mass spectrometer detector (model: LCMS-8045, Shimadzu, Kyoto, Japan). 

A model KJO-4282 Guard Cartridge System and a Lux Cellulose-2 (LC-2) chiral column with a cellulose tris (3-chloro-4-methylphenylcarbamate) stationary phase, as well as a Lux Cellulose-3 (LC-3) chiral column with a cellulose tris(4-methyl benzoate) from Phenomenex Co. (Torrance, CA, USA), were used in chiral chromatographic separations.

All incubations were performed in a dedicated incubator, including model Incubators 1000 and Unimax 1010, and were purchased from Heidolph (Schwabach, Germany) with controlled temperature and rotation (250 rpm). Each piece of glass used was dried in an oven overnight before being cooled with a stream of nitrogen.

### 3.3. Chromatographic Conditions

Various chromatographic conditions were investigated to optimize the chiral separations of all reagents, e.g., enantiomers of the racemic β-blocker building block and its esters. During the studies, four chiral columns were investigated, e.g., Lux^®^ Cellulose-1 (Cellulose tris(3,5-dimethylphenylcarbamate), Lux^®^ Cellulose-2 (Cellulose tris(3-chloro-4-methylphenylcarbamate), Lux^®^ Cellulose-3 (Cellulose tris(4-methylbenzoate), and Lux^®^ Amylose-1 (Amylose tris(3,5-dimethylphenylcarbamate), with various mobile phase compositions containing acetonitrile, methanol, and diethylamine; methanol, acetonitrile and formic acid methanol, isopropanol and diethylamine; methanol, isopropanol and formic acid; acetonitrile, isopropanol, and diethylamine, or acetonitrile, isopropanol and formic acid. Finally, the baseline chiral separations of the enantiomer-studied analytes were accomplished using the Lux Amylose-1 chiral column. The mobile phase composition for the chiral separation of studied analytes was the acetonitrile, isopropanol, and diethylamine mixture at a volumetric ratio of 93.5/6.5/0.1.

**Figure 4:**
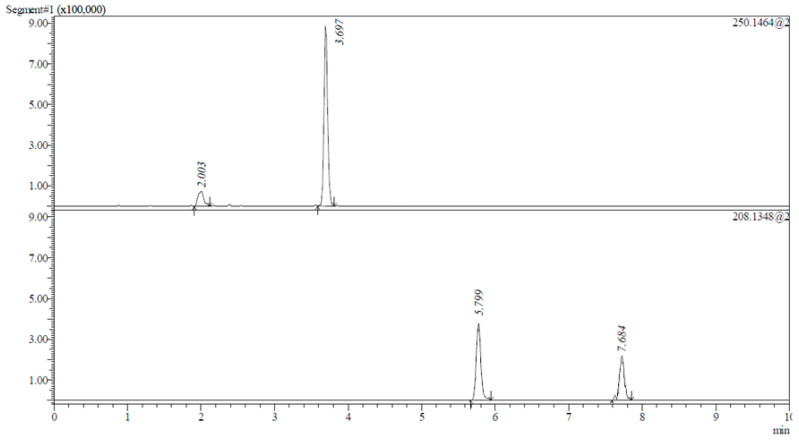
Chromatogram showing the peaks of kinetic resolution products ((*R*)-1-(isopropylamino)-3-phenoxy-2-propanol acetate (R_t_ = 2.003 min) and (*S*)-1-(isopropylamino)-3-phenoxy-2-propanol acetate (R_t_ = 3.697 min)) and substrates ((*R*)-1-(isopropylamino)-3-phenoxy-2-propanol (R_t_ = 5.799 min) and (*S*)-1-(isopropylamino)-3-phenoxy-2-propanol acetate (R_t_ = 7.684 min)) obtained by LCMS-IT-TOF. Analysis conditions: Lux^®^ 5 µm Amylose-1 column. Mobile phase: acetonitrile/isopropanol/diethylamine 93.5/6.5/0.1, flow rate 0.8 mL/min, injection volume 10 µL, *m*/*z* = 250.1464 and *m*/*z* = 208.134.

In optimized chromatographic conditions, the mobile phase flow rate was set at 0.8 mL/min to derive a proper resolution. An IT-TOF mass spectrometer in scan mode was utilized to detect chiral compounds. The retention time for (*R*)-1-(isopropylamine)-3-phenoxy-2-propanol acetate was t_R_ = 2.003 min, and for (*S*)-1-(isopropylamine)-3-phenoxy-2-propanol acetate it was t_R_ = 3.697 min, whereas for (*R*)-1-(isopropylamine)-3-phenoxy-2-propanol it was t_R_ = 5.799 min, and for (*S*)-1-(isopropylamine)-3-phenoxy-2-propanol it was t_R_ = 7.684 min, as is shown in Figure 4.

Using equations described by Chen et al. [34,35] based on peak areas from chromatograms of enantiomers of clopidogrel carboxylic acid and clopidogrel, it was possible to determine the conversion and optical purity of both substrates and products, as well as the enantioselectivity of the enzyme-catalyzed biotransformation that was carried out.

### 3.4. Chemical Acetylation of 1-(Isopropylamine)-3-Phenoxy-2-Propanol

**Figure 5:**
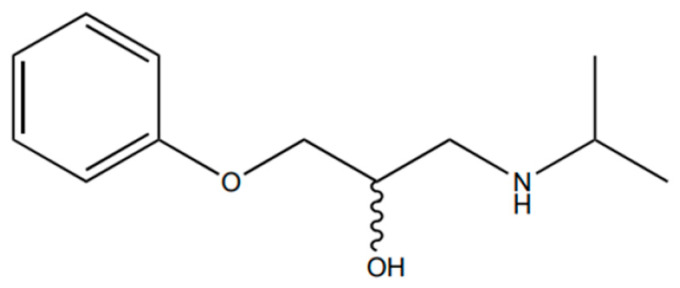
The chemical structure of the β-blocker building block is racemic 1-(isopropylamine)-3-phenoxy-2-propanol.

**Figure 6:**
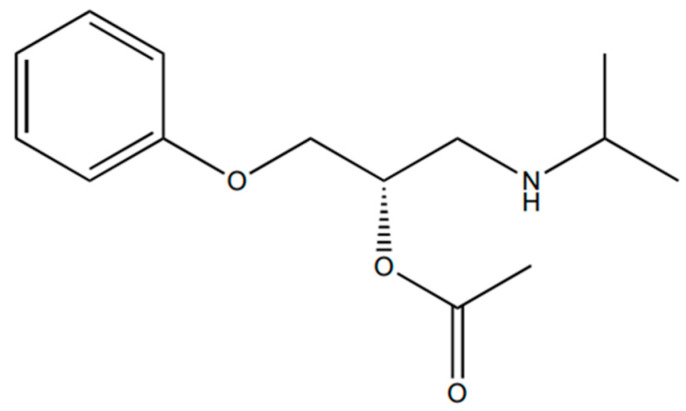
Chemical structure of β-blocker building block derivative (*S*)-1-(isopropylamine)-3-phenoxy-2-propanol acetate.

The 1-(isopropylamine)-3-phenoxy-2-propanol (Figure 5) was acetylated according to the previously reported methodology, with a few modifications [36]. Briefly, the racemic compound (0.002 g; 0.00957 mmol) was refluxed with dichloromethane (10 mL), and acetyl chloride (8 µL; 0.076 mmol) was very slowly added. After that, the reaction mixture was incubated at 30 °C for two hours and washed successively with equal volumes of saturated aqueous sodium bicarbonate and brine. The organic layer was collected and evaporated to dryness under a vacuum. Finally, the derivation of 1-(isopropylamine)-3-phenoxy-2-propanol (Figure 6) was used as a standard to establish an optimal chromatographic method, which allowed for quantitatively determining the racemic atenolol and its acetylated form.

### 3.5. Kinetic Resolution of β-Blockers Building Block

**Figure 7:**
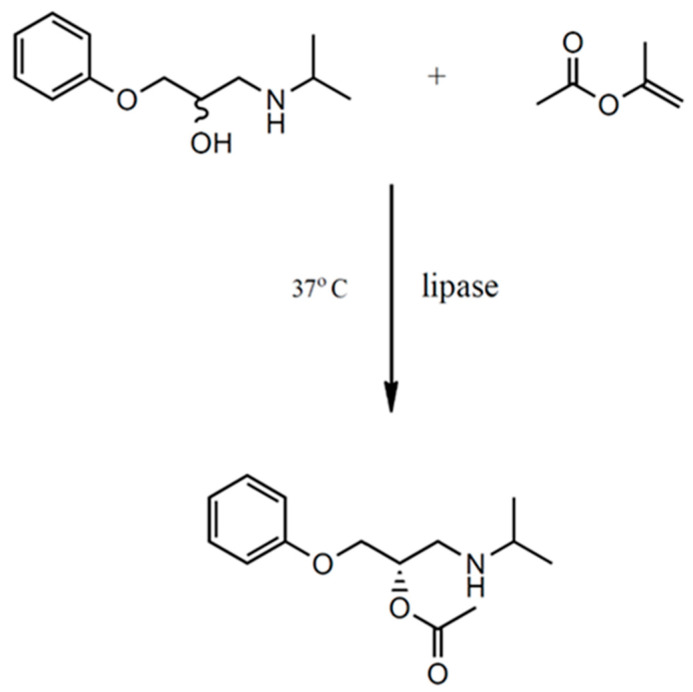
Scheme of kinetic resolution of the β-blockers building block.

The stereoselective building-block biotransformation of the β-blocker was performed to obtain pure enantiomers, such as 1-(isopropylamine)-3-phenoxy-2-propanol using ionic liquids and lipases from *Candida rugosa* in native form in a two-phase catalytic system. A schematic of the reaction that took place is shown in Figure 7.

To assess the kinetic resolution, 2 mg racemic mixtures of 1-(isopropylamine)-3-phenoxy-2-propanol were placed in 24 glass-stoppered flasks (Erlenmayer flasks) of 50 mL each. Dichloromethane (4 mL) was added as a reaction medium to 12 flasks, and 4 mL of toluene was added to the remaining 12 flasks. Each of the 12 reaction systems was divided into two parts. Isopropenyl acetate (1 µL) was added to 6 flasks containing dichloromethane, and vinyl acetate (1 µL) was added to the remaining six flasks as an acylating agent. The same steps were followed for the 12 flasks containing toluene. The groups of 6 flasks were then again divided into halves. The top 3 from each group were added with lipase from *Candida rugosa* OF (10 mg) and the remaining 3 were added with lipase from *Candida rugosa* MY (10 mg). A total of 24 reaction systems were obtained, consisting of 3 groups of 8 identical reaction systems each. Group I was left without the addition of ionic liquid, while the ionic liquid 1-ethyl-3-methylimidazolium tetrafluoroborate [EMIM][BF_4_] was added to all reaction systems in group II, and the ionic liquid 1-ethyl-3-methylimidazolium trifluoromethane sulfonate ([EMIM][MSF_3_]) was added to group III. The prepared reaction systems were incubated in a shaker incubator for 240 h at 37 °C, at 250 rpm. The compositions of the qualitative individual reaction systems 1 to 24 are shown in the table (Table 1).

**Figure 8:**
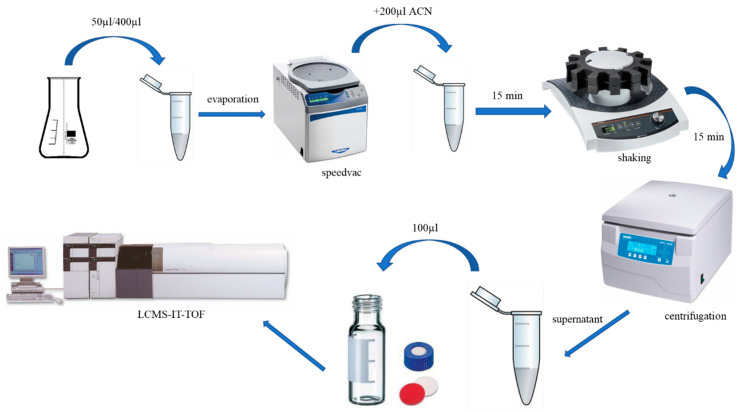
Scheme of the sampling procedure.

The resulting 24 reaction systems were sampled at equal intervals every 24 h. The total incubation time was 240 h. The sampling procedure consisted of the following steps (Figure 8): taking 400 µL of test solution from reaction systems containing no ionic liquid (1–8) and 50 µL of test solution from reaction systems containing ionic liquids (9–24); the samples were placed in a speed vac until the volatile solvents were completely evaporated; 200 µL of acetonitrile was added to each of the 24 samples, set in a shaker for 15 min and centrifuged for 15 min at 20 °C at 18,000 rpm in the next step; the supernatant obtained after centrifugation was then transferred to the glass insert vials and analyzed via UPLC-IT-TOF.

## 4. Discussion

To discuss the results, a literature analysis of published items, including oral presentations, was conducted. Scientific databases of full-text articles such as PubMed, Web of Knowledge, and Google Scholar were used during the analyses. Time criteria did not restrict analyses. Searches were based on combinations of the following keywords in English, combined using logical operators (and): atenolol, propranolol, sotalol, pindolol, beta-blocker, β-blocker, and kinetic resolution.

**Figure 9:**
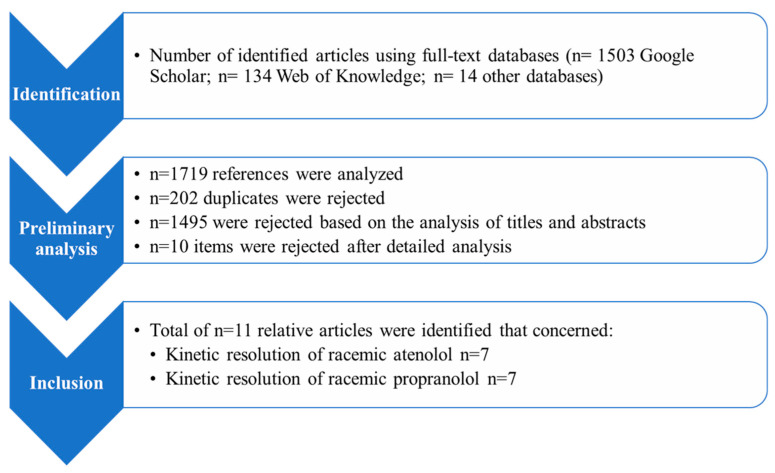
Literature analysis scheme.

All the literature items were initially included in the literature analysis. No language restrictions were applied in assessing experimental studies describing methods for kinetic resolution of β-blockers. Ultimately, 1719 literature items were identified through the analysis, 134 in Web of Knowledge, 1503 in Google Scholar, and 14 in PubMed. Duplicate/multiple literature items and papers not containing relevant information were excluded. Finally, 11 original scientific articles related to the enzymatic kinetic resolution of racemic atenolol and propranolol were considered eligible for inclusion in the literature study, as shown in Table 3. A detailed scheme of the literature analysis is presented in Figure 9.

Of all the studies conducted on the kinetic resolution of β-blockers, racemic atenolol is the compound most commonly biotransformed and described by researchers. The identified studies show that the kinetic resolution of (*R*, *S*) also describes propranolol. However, no other scientific papers focused on the kinetic resolution of other β-blockers (Table 3).

An analysis of the scientific papers on the kinetic separation of (*R*, *S*) atenolol shows that researchers tested different catalytic systems. However, the enantioselective biotransformation of racemic atenolol using toluene or [EMIM][BF_4_] as the reaction medium, isopropenyl acetate or vinyl acetate as the acetylating agent, and *Candida rugosa* or *Candida antarctica* lipase yielded the highest enantioselectivity [31,37,38,39,40,41,48].

Similarly, according to the identified articles, for the kinetic separation of (*R*, *S*) propranolol, catalytic systems containing toluene as reaction medium, vinyl acetate or isopropenyl acetate as an acetylating agent, and lipase from *Candida rugosa* or *Candida antarctica* afforded enantiomerically pure propranolol esters [42,43,45,47].

Therefore, it must be objectively stated that all the systems tested within the scope of the present work yield data consistent with those in the literature. Moreover, despite the previously cited limitations of the studies performed, which should be eliminated in the case of further research tasks, the results obtained for the enantioselective biotransformation of the building block β-blockers can be considered enantioselective and correspond to those obtained by other researchers in other external projects on the enzymatic kinetic separation of racemic propranolol and atenolol.

## 5. Conclusions

The optimized kinetic resolution of 1-(isopropylamine)-3-phenoxy-2-propanol showed the most optimal reaction conditions. The enantioselective biotransformation of racemic 1-(isopropylamine)-3-phenoxy-2-propanol using [EMIM][BF_4_] and toluene as a two-phase reaction medium, isopropenyl acetate as an acylating agent, and *Candida rugosa* MY lipase yielded the highest enantioselective excess of product, which was ee_p_ = 96.2%. The results show the most efficient conversion (C = 28.2%) and the best enantioselectivity (E = 67.5). The presence of [EMIM][BF_4_] favorably affected the biotransformation of 1-(isopropylamine)-3-phenoxy-2-propanol, as the value of enantioselectivity was more than two times higher when the ionic liquid was added to the reaction mixture compared to the system without the ionic liquids.

## Figures and Tables

**Table 1:** Composition of tested reaction systems.

No	Acetylating Agent	Reaction Medium	Lipase	Ionic Liquid
1	vinyl acetate	toluene	OF	n/a
2	vinyl acetate	toluene	MY	n/a
3	isopropenyl acetate	toluene	OF	n/a
4	isopropenyl acetate	toluene	MY	n/a
5	vinyl acetate	dichloromethane	OF	n/a
6	vinyl acetate	dichloromethane	MY	n/a
7	isopropenyl acetate	dichloromethane	OF	n/a
8	isopropenyl acetate	dichloromethane	MY	n/a
9	vinyl acetate	toluene	OF	[EMIM][BF_4_]
10	vinyl acetate	toluene	MY	[EMIM][BF_4_]
11	isopropenyl acetate	toluene	OF	[EMIM][BF_4_]
12	isopropenyl acetate	toluene	MY	[EMIM][BF_4_]
13	vinyl acetate	dichloromethane	OF	[EMIM][BF_4_]
14	vinyl acetate	dichloromethane	MY	[EMIM][BF_4_]
15	isopropenyl acetate	dichloromethane	OF	[EMIM][BF_4_]
16	isopropenyl acetate	dichloromethane	MY	[EMIM][BF_4_]
17	vinyl acetate	toluene	OF	[EMIM][MSF_3_]
18	vinyl acetate	toluene	MY	[EMIM][MSF_3_]
19	isopropenyl acetate	toluene	OF	[EMIM][MSF_3_]
20	isopropenyl acetate	toluene	MY	[EMIM][MSF_3_]
21	vinyl acetate	dichloromethane	OF	[EMIM][MSF_3_]
22	vinyl acetate	dichloromethane	MY	[EMIM][MSF_3_]
23	isopropenyl acetate	dichloromethane	OF	[EMIM][MSF_3_]
24	isopropenyl acetate	dichloromethane	MY	[EMIM][MSF_3_]

**Table 2:** Kinetic resolution parameters of racemic 1-(isopropylamine)-3-phenoxy-2-propanol depending on the applied reagents after 24 h; ee_s_—enantiomeric excess of substrates; ee_p_—enantiomeric excess of products; C—conversion; E—enantioselectivity.

No	ee_s_	ee_p_	C	E
1	5.3%	62.2%	7.8%	5.5
2	1.0%	52.4%	1.9%	3.2
3	8.1%	58.0%	12.2%	4.1
4	24.5%	91.6%	21.1%	28.9
5	2.1%	60.7%	3.3%	4.2
6	2.3%	76.1%	2.9%	7.5
7	14.0%	44.4%	24.0%	3.0
8	10.1%	37.0%	21.4%	2.4
9	5.4%	64.0%	7.7%	4.8
10	1.0%	55.1%	1.9%	3.5
11	8.3%	59.1%	12.3%	4.2
12	**28.2%**	**96.2%**	**22.7%**	**67.5**
13	2.1%	62.5%	3.3%	4.4
14	2.3%	79.9%	2.8%	9.1
15	14.4%	45.3%	24.1%	3.1
16	10.6%	38.1%	21.7%	2.5
17	4.6%	65.9%	6.5%	5.1
18	0.9%	57.8%	1.5%	3.8
19	6.7%	50.2%	11.8%	3.2
20	22.3%	79.8%	21.8%	11.1
21	1.8%	50.6%	3.5%	3.1
22	1.9%	63.1%	3.0%	4.5
23	11.7%	38.5%	23.3%	2.5
24	8.4%	31.7%	20.9%	2.1

**Table 3:** List of studies on the enzymatic kinetic separation of racemic atenolol and propranolol.

Author	Racemic Compound	Literature	E-Ratio	Lipase	Acetylating Agent	Reaction Medium
Agustian et al.	(*R*, S)-atenolol	[37]	17	*Pseudomonas fluorescence*	Vinyl acetate	Tetrahydrofuran
Barbosa et al.	(*R*, S)-atenolol	[38]	65	*Candida antarctica* B	Vinyl acetate	Toluene, Hexane
Dvivedee et al.	(*R*,S)-atenolol	[31]	210	*Candida antarctica* A, *Psudomonas cepacia* (PCL), *Mucor meihei* (MML), *Candida rugosa* (CRL L8525), *Candida rugosa* (CRL L1754), *Candida rugosa* (CRL 62316), *Candida cylindracea* (CCL), *Aspergillus niger* (ANL), *porcine pancreas lipase* (PPL), *lipase AY* “*Amano 30*” (CRL).	Vinyl acetate	[EMIM][BF_4_] and toluene, acetonitrile
Lund et al.	(*R*, S)-atenolol	[39]	278	*Candida antarctica* B	Vinyl butanoate	Toluene
Sikora et al.	(*R*,S)-atenolol	[36,40,41]	67	*Candida rugosa* (OF), *Candida rugosa* (MY), *Alcaigenes fecalis* (QLM), *Pseudomonas strutzeri* (TL), *Candida antarctica* (CALBY), *Aspergillus niger*, *Burkholderia cepacia*, *Burkoholderia cepacia* (SL) *Alcaigenes sp.* (PL).	Isopropenyl acetate, vinyl acetate	Toluene, acetonitrile, tetrahydrofuran, chloroform
Chiou et al.	(*R*, S)-propranolol	[42]	21	*Burkholderia cepacia* (Amano PS), *Candida rugosa* MY, *Candida rugosa* OF, *Amano L10*, *Amano AP15*, *Amano D20*, *Candida rugosa* (Sigma)	Isopropenyl acetate, vinyl acetate	Toluene, chloroform, isopropyl ether, tetrahydrofuran, acetonitrile
Barbosa et al.	(*R*, S)-propranolol	[43]	57	*Candida antarctica* B	Vinyl acetate	Toluene
Bornscheuer et al.	(*R*, S)-propranolol	[44]	-	*Candida antarctica* B	Vinyl acetate, cyclohexyl acetate	Dodecane
Escorcia et al.	(*R*, S)-propranolol	[45,46]	63	*Candida antarctica* B	Vinyl acetate	The mixture of toluene and methanol
Gamboa-Velazquez et al.	(*R*, S)-propranolol	[47]	259	*Candida antarctica* B	Not applicable	The mixture of dioxane and water

## Data Availability

Data are contained within the article.
